# Extensive Synovial Osteochondromatosis Secondary to Avascular Necrosis of the Femoral Head: A Case of Multifocal Extra-articular Involvement

**DOI:** 10.7759/cureus.91715

**Published:** 2025-09-06

**Authors:** Quang Dai La, Aiman Baloch, Muhammad Ayub, Sobia Ahmed, Han B La, Rizwana Rahman, Francis Pryor, Mahwash Mansoor

**Affiliations:** 1 Biology, Texas A&M University, College Station, USA; 2 Medicine, The Innovative STEMagazine, College Station, USA; 3 Medicine, Mekran Medical College Turbat, Balochistan, PAK; 4 Radiology, Bolan Medical Complex Hospital, Quetta, PAK; 5 Medicine, Texas A&M College of Medicine, College Station, USA; 6 Medicine, Lake Erie College of Osteopathic Medicine, Erie, USA; 7 Diagnostic Radiology, Bolan Medical College Quetta, Quetta, PAK

**Keywords:** avascular necrosis, ct imaging, hip joint, joint degeneration, loose bodies, musculoskeletal pathology, orthopedic surgery, osteoarthritis, secondary soc, synovial osteochondromatosis

## Abstract

Synovial osteochondromatosis (SOC) is an uncommon benign disorder of synovial metaplasia, characterized by cartilaginous or osseous loose bodies within the joint, bursa, or tendon sheath. We report a case of a 40-year-old female who presented initially with hip pain and limitation of motion on her right side. Imaging revealed avascular necrosis (AVN) of the femoral head along with depicted early SOC. The patient had significant symptom relief with conservative management. However, at follow-up, imaging consistently demonstrated significant progression of SOC with numerous calcified loose bodies intra- and extra-articularly involving adjacent muscle groups. There were also moderate findings of secondary osteoarthritis of the hip noted. The patient was scheduled for open surgical synovectomy. This case illustrates the aggressive nature of secondary SOC within the setting of the necrotic femoral head and calls for early imaging and surgical planning within the complex arena of joint disorders.

## Introduction

Synovial osteochondromatosis (SOC) is an unusual benign condition characterized by metaplastic changes in the synovium to osteocartilaginous nodules that can become detached and develop into loose bodies in joints, bursae, or tendon sheaths [1]. Primary SOC is idiopathic, while secondary SOC typically occurs in the context of other identified joint pathology, including osteoarthritis, trauma, or avascular necrosis (AVN) [2]. The incidence rate of SOC in the hip joint in primary or secondary forms is comparatively low among larger joints, and extra-articular extension, such as pericapsular and periarticular musculature, is infrequent [3]. 

Avascular necrosis of the femoral head contributes to abnormal loading of the hip joint and can lead to early degenerative osteoarthritis, which may facilitate an inciting factor in developing secondary SOC [4]. There are numerous case studies reporting SOC as a cause-and-effect after development of AVN-induced hip degeneration that resulted in symptomatic worsening and subsequent loss of hip range of motion with degenerative change [5]. Imaging typically shows multiple calcified loose bodies in the joint and perhaps in soft tissues; CT and MRI can allow for a complete understanding of the disease extent intra- and extra-articularly [6].

Management is usually surgical to remove the loose bodies and perform a synovectomy. If the disease is not extensively involved, an arthroscopic approach can be satisfactory, while an open surgical approach is warranted if the disease is extensive or extra-articular [7]. In the instance of hip SOC with AVN, surgical evaluation must be extensive to be able to appropriately address the conditions and preserve joint function [8]. We present the case of a 40-year-old female with right femoral head AVN and extensive communication of intra- and extra-articular SOC, inclusive of multiple periarticular muscle involvements identified in imaging, that was treated surgically.

This case was presented as a poster at the 40th Annual Radiological Society of Pakistan (RSP) Radiological Conference in Collaboration with the Royal College of Radiologists (UK) between November 8th and 10th, 2024.

## Case presentation

**Figure 1:**
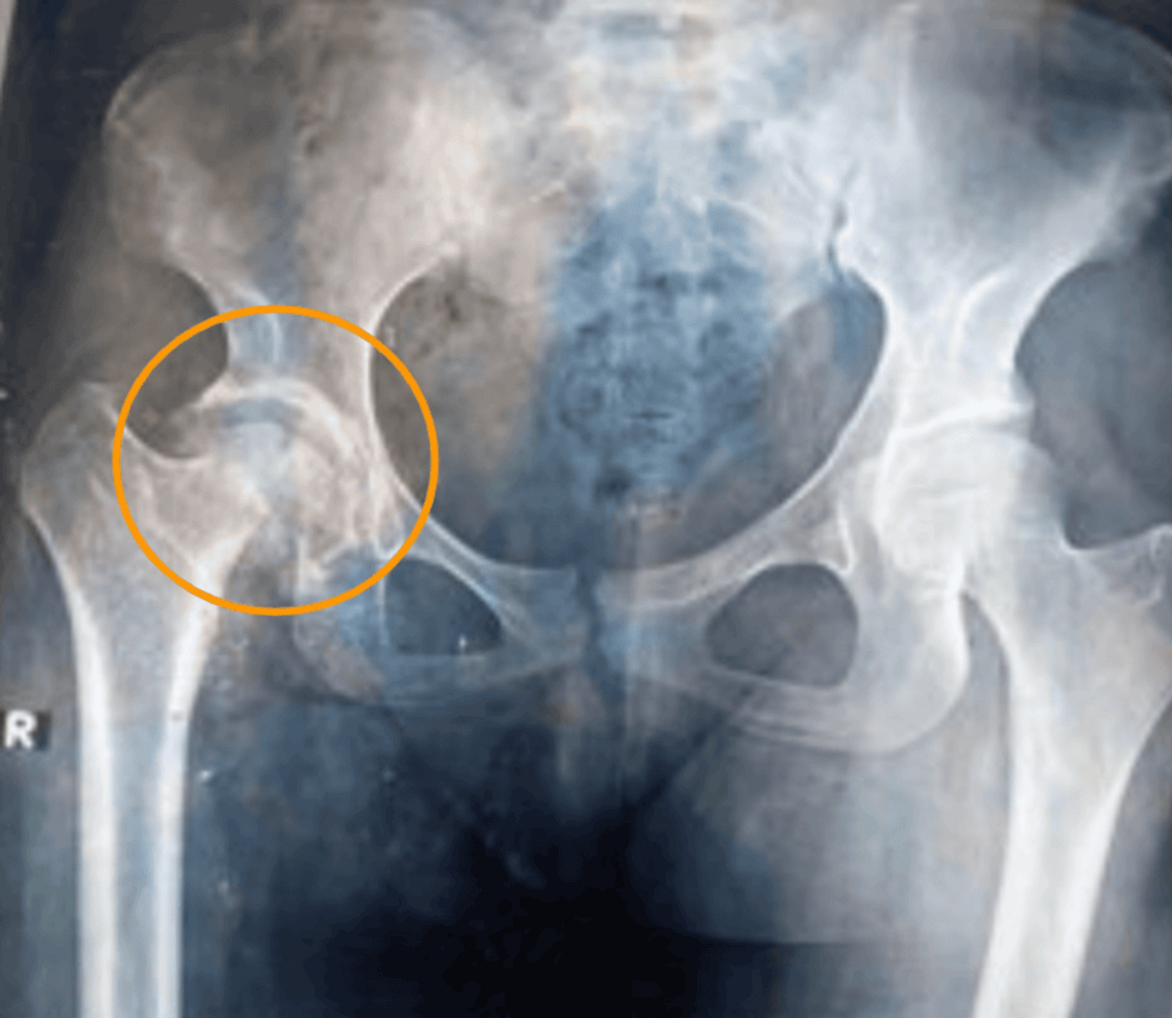
Frontal radiograph of pelvis including both proximal femurs. Avascular necrosis of right femoral head with reduced head volume and widened acetabulum. Early development of synovial osteochondromatosis was also evident showing few calcified loose bodies. The yellow circle encompasses the AVN of the right femoral head with reduced head volume and widened acetabulum. Early development of synovial osteochondromatosis was also evident, showing a few calcified loose bodies. AVN: Avascular necrosis

A 40-year-old female presented to the orthopedic clinic with complaints of right hip pain and limited range of motion. The initial radiographic assessment revealed AVN of the right femoral head, demonstrated by volume loss and deformity of the femoral head, along with acetabular widening. As well, the beginnings of synovial osteochondromatosis were noted, with calcified intra-articular loose bodies identified on imaging (Figure 1). The patient was initially conservatively managed by the orthopedic team.

**Figure 2:**
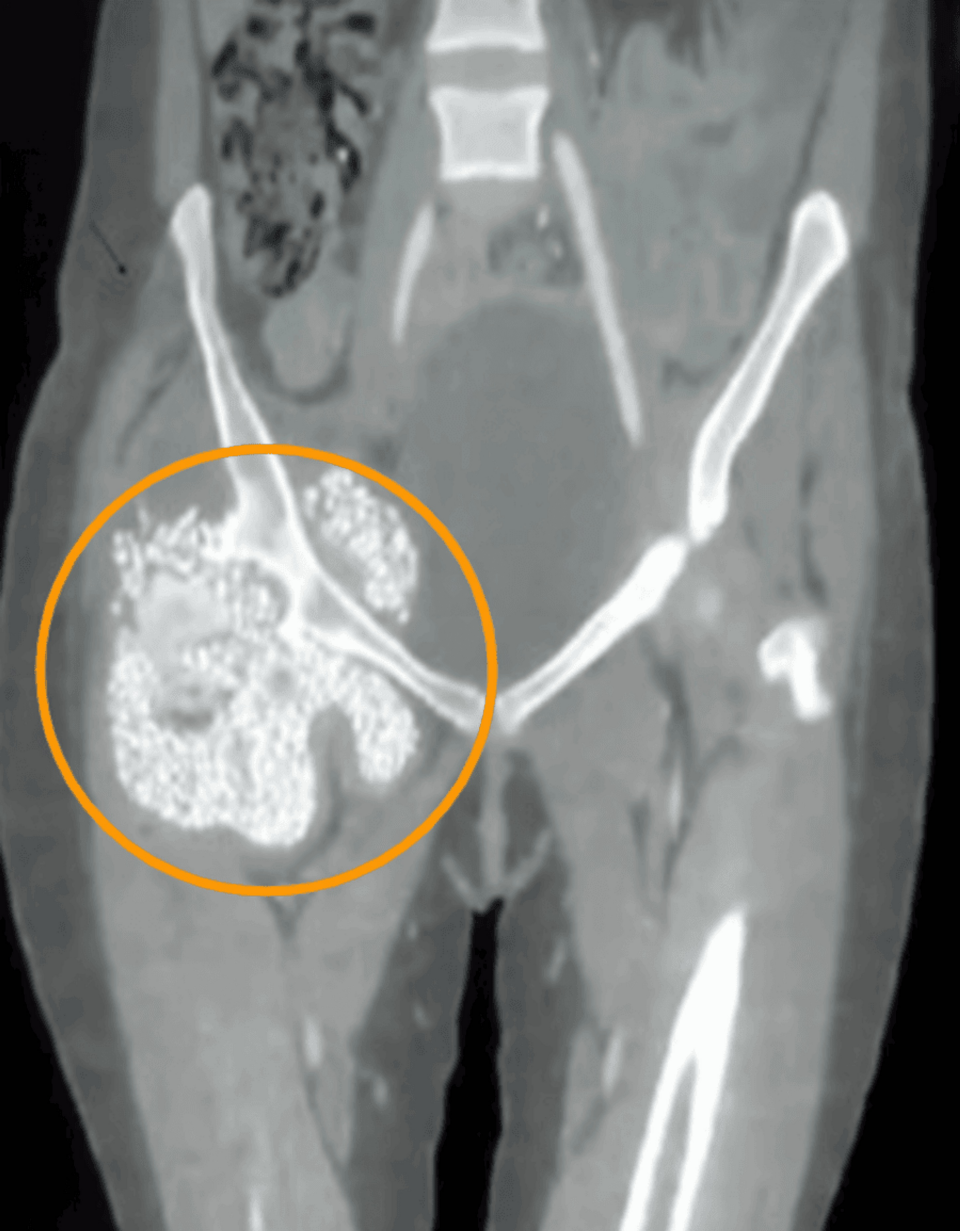
CT of the pelvis, including the bilateral proximal lower limb bone window Extensive encapsulated synovial osteochondromatosis with innumerable intra and extra-articular osseous and osseocartilaginous loose bodies are observed within the yellow circle. A few of these calcific foci were enlarged, involving the iliopsoas, gluteal, pectineus, obturator externus, and adductor muscles of the same side.

**Figure 3:**
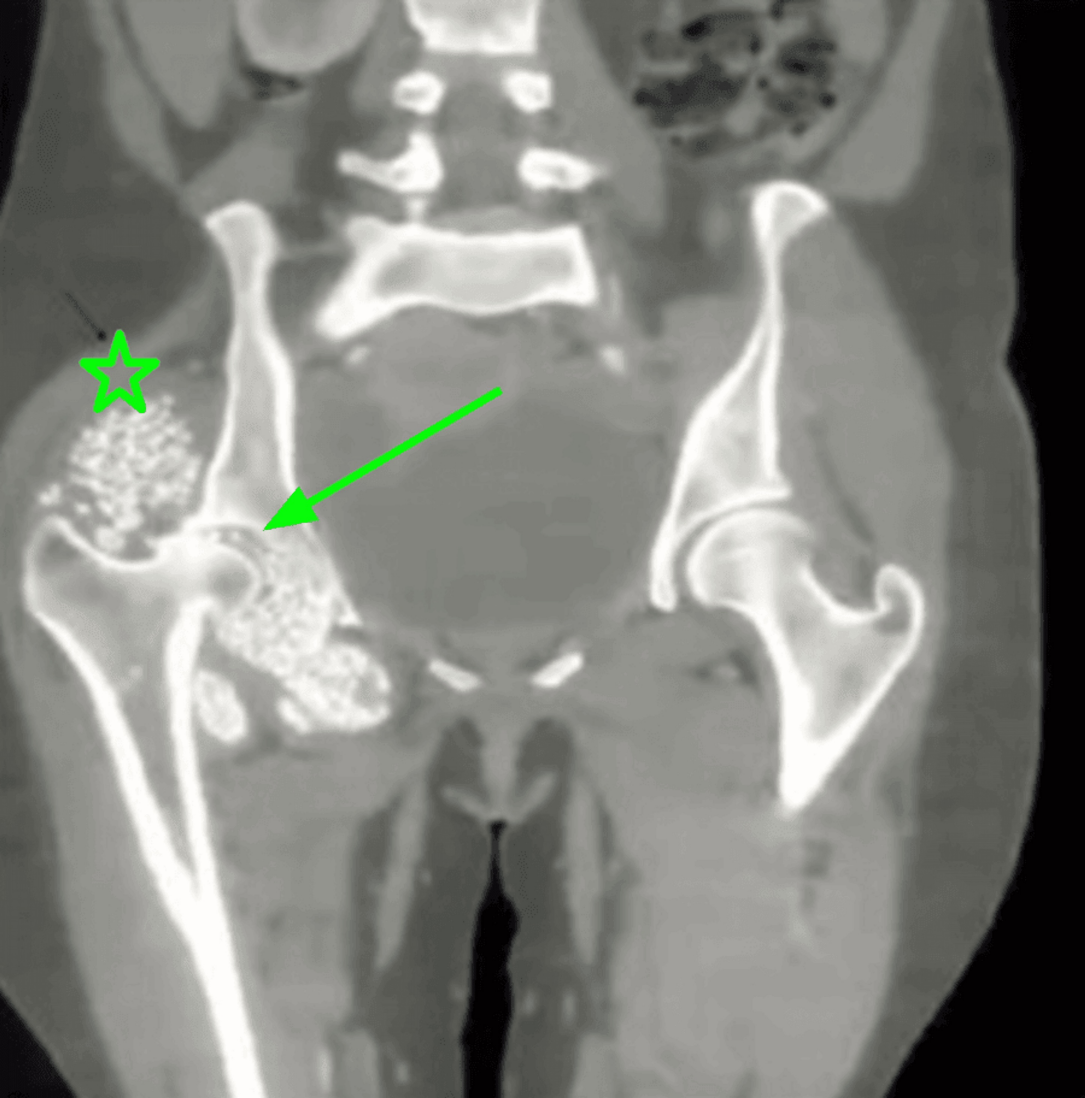
CT of the pelvis, including the bilateral proximal lower limb bone window Arrow: Signs of development of secondary osteoarthritis were also evident by articular sclerosis of the acetabulum with subarticular cystic areas, irregularities, and small erosions; Star: Gluteus medius in comparison to the left side showed a reduced bulk likely secondary to atrophy

During the next four months, the patient experienced gradual expansion of the right hip area. Follow-up imaging with radiographs and CT scans revealed marked progression of disease. A large degree of encapsulated synovial osteochondromatosis with innumerable intra- and extra-articular osseous and osseocartilaginous loose bodies involving the iliopsoas, gluteal, pectineus, obturator externus, and adductor muscles on the right was observed (Figures 2-3). Secondary osteoarthritic changes included irregularities of the articular surface, subarticular cystic changes, sclerosis, and erosions of the right acetabulum. Follow-up imaging also showed atrophy of the right gluteus maximus muscle. These findings were consistent with a late sequela of AVN of the femoral head complicated by secondary osteoarthritis and extensive synovial osteochondromatosis. After further radiographic imaging, the patient was listed for open surgical intervention by the orthopedic team.

## Discussion

Synovial osteochondromatosis of the hip is a rare benign condition that is related to synovial metaplasia and the development of loose bodies in and out of the joint capsule. Primary SOC arises without any apparent cause, while secondary SOC, on the other hand, is associated more with previous joint pathology, such as osteoarthritis, trauma, or AVN of the femoral head [8,9]. Primary SOC of the hip can be treated arthroscopically, but complex or extensive cases, particularly those with extra-articular disease, may require open synovectomy and removal of loose bodies [5,8].

In a case reported by van der Valk et al., giant synovial chondromatosis of the hip was identified with extra-articular extension into the iliopsoas bursa, which was surgically excised via an open procedure [9]. This is similar to the current case, which showed extensive intra- and extra-articular loose bodies within the iliopsoas, gluteal, pectineus, obturator externus, and adductor muscles on CT imaging. This indicates significant disease progression.

Takeda et al. noted that the complete removal of loose bodies using open or mechanically assisted arthroscopic approaches is critical to preventing recurrence [10]. Similarly, Lin et al. demonstrated that arthroscopic-assisted mini-open surgery creates a successful synovectomy and loose body removal without joint dislocation or avascular necrosis [8]. In this case, imaging revealed secondary osteoarthritis with degenerative changes, confirming the utility of a holistic surgical approach to treat SOC and AVN changes together.

Arthroscopic studies have shown variable recurrence rates (7% to 39%), with the presence of loose bodies after surgery being positively correlated with recurrence, and thus, wide capsulectomy and synovectomy have been recommended in an attempt to reduce recurrence [8,11]. While open approaches are more invasive, they may be more appropriate for multifocal processes, such as the indices of multi-compartment involvement present here, than the arthroscopic methodologies.

## Conclusions

This case demonstrates secondary SOC of the hip resulting from AVN with extensive and severe intra- and extra-articular disease. Imaging is important for surgical planning. The literature suggests that either an open or combined open-arthroscopy approach would be preferable, as this allows the surgeon to safely complete a synovectomy and remove loose bodies, thus minimising recurrence and maintaining hip mobility.
